# Neuronal cytoplasmic inclusion bodies in the brain of Lagotto Romagnolo dogs: A qualitative and quantitative histologic evaluation

**DOI:** 10.1177/03009858241300555

**Published:** 2024-12-09

**Authors:** Sini Peura, Elina Kiiskinen, Tarja S. Jokinen, Anna-Maija K. Virtala, Pernilla Syrjä

**Affiliations:** 1 Department of Veterinary Biosciences, Faculty of Veterinary Medicine, University of Helsinki, Helsinki, Finland; 2 Department of Equine and Small Animal Medicine, Faculty of Veterinary Medicine, University of Helsinki, Helsinki, Finland

**Keywords:** dog, Lagotto Romagnolo, neurodegeneration, neuronal cytoplasmic inclusion body, proteinopathy, PTAH-positive inclusion

## Abstract

Four neurologic diseases affect dogs of the Lagotto Romagnolo (LR) breed, namely benign familial juvenile epilepsy (BFJE), vacuolar storage disease, and 2 forms of cerebellar cortical degeneration. Intraneuronal inclusion bodies in cerebellar Purkinje cells were first described in the BFJE phenotype. Upon further characterization of these diseases, similar inclusions were also noted in the brain of LRs used as controls. This study investigated the clinical, histologic, and electron microscopic findings in 23 LRs to determine the nature of their neuronal inclusions and whether the presence, distribution, or number of inclusions is associated with neurologic signs. Electron microscopy of the inclusions revealed a cytoplasmic aggregate without a limiting membrane. The inclusions appeared proteinaceous on histochemical staining and positive on phosphotungstic-acid-hematoxylin (PTAH) stain for proteins rich in basic amino acids. Markers of commonly known proteinopathies of humans (ubiquitin, p62, LC3, α-synuclein, and β-amyloid) were not detected in the inclusions when assessed by immunohistochemistry. The overall presence of inclusion bodies was not significantly associated with the dog’s neurologic status. The results show an association between inclusions in the cerebral cortex and an absence of clinical neurologic disease in LRs. There was no significant difference in the quantitative inclusion body burden when compared in LRs with or without neurologic signs. Although PTAH-positive proteinaceous neuronal inclusions are a common finding in LRs regardless of neurologic signs, these inclusions may be a protective response when present in the cerebral cortex.

Noninfectious neuronal cytoplasmic inclusion bodies are intracellular aggregates consisting of misfolded or accumulated subcellular components (often protein) that have not been eliminated by protective cellular degradative systems. Neuronal cytoplasmic inclusion bodies are part of the hallmark histopathology in several neurodegenerative diseases in humans, including α-synuclein containing Lewy-bodies in Parkinson’s disease (PD) or aggregates of Huntingtin exon 1-encoded polyglutamine fragments in Huntington’s disease.^1,3,21^ Alternatively, inclusions and aggregates may contain marker proteins from various cellular pathways, indicating which pathogenic mechanisms are involved in formation of the aggregates. Ubiquitin is a known marker of disturbed proteasomal or autophagic clearance in neurons, whereas the autophagic cargo marker p62 and autophagosomal membrane marker LC3 specifically indicate aberrant autophagy in the cell.^17,19^ Protein accumulation within the amyloidogenic pathway, typical for Alzheimer’s disease (AD), can be assessed by visualizing β-amyloid deposits.^5^

Intracytoplasmic inclusions may contribute to neurodegeneration by interfering with normal intracellular metabolism and transport, thus further stressing the cells’ protective mechanisms.^21^ However, the formation of inclusions may also represent a protective cellular response in neurons, where toxic or reactive substances are isolated from the intracellular space.^3^ Therefore, the role of cytoplasmic inclusions in pathogenesis should be assessed specifically for each disease.

The dog is increasingly being used as a spontaneous model of neurodegenerative disease.^2,31^ With its shorter lifespan, relatively similar living environment, and similar pathogenic mechanisms that affect the brain as in humans, the dog has brought valuable insights in this field of research. Neurodegeneration due to disturbed autophagy that is detectable histologically with markers comparable with those used in humans has been observed in Lagotto Romagnolo (LR), Spanish waterdog, and Gordon setter breeds.^1,12,16,26^ Classical intracytoplasmic Lewy bodies have not been described in dogs in conjunction with signs of Parkinsonism, and polyglutamine aggregates or phenotypes modeling Huntington’s disease have not been reported. The LR is an old Italian dog breed, originally used as a truffle dog. Four neurological diseases occur in LR dogs, namely benign familial juvenile epilepsy (BFJE), 2 types of cerebellar cortical degeneration, and a vacuolar storage disease.^14–16^ Eosinophilic, round to elongated, 2 to 5 µm in diameter, neuronal cytoplasmic inclusion bodies were first described in Purkinje cells as part of the BFJE phenotype in LRs.^14^ A viral infectious etiology was excluded in these cases and has also been considered by other investigators in cases with similar findings.^8,13,14,18,20^ During postmortem investigations of LRs in the study of BFJE and vacuolar storage disease, we noted comparable neuronal inclusions also in control LRs. Therefore, we sought to define the content of the inclusions and to investigate if their occurrence or distribution pattern is associated with the dog’s neurologic status. The final aim of this study was to clarify if the inclusion bodies in LR neurons are an incidental or a pathologic finding.

## Material and Methods

### Material

Clinicopathologic records and histologic samples of LRs euthanized and autopsied at the Faculty of Veterinary Medicine, University of Helsinki from 2012 to 2018, were retrieved for this retrospective study. The LRs >3 months of age with documented clinical and neurologic examinations before euthanasia were included in the study. Only LRs with histologic samples available of all brain regions of interest (nucleus caudatus, motor cortex, Ammon’s horn, geniculate nuclei of the thalamus, substantia nigra, cerebellar cortex with Purkinje cells, and medulla oblongata) were included. The clinical and neurologic findings of all dogs were retrieved.

### Ethics Statement

The autopsied LRs were privately owned pets and were undergoing euthanasia and autopsy at owner request either due to progressive neurologic decline or for other reasons. The animals were submitted for autopsy with signed consent of the owner to allow for use of the carcass for research purposes. The use of surplus biologic samples for research purposes was approved by the Viikki Campus Research Ethics Committee, University of Helsinki (Statement 13/2020). The Faculty of Veterinary Medicine, University of Helsinki, Section for Pathology and Parasitology withholds all further rights to the histology specimens submitted to analysis or obtained at postmortem. This faculty policy is informed to clients in writing on the postmortem submission form.

### Histochemistry and Immunohistochemistry of the Inclusion Bodies

The study material for the qualitative study of the inclusion bodies consisted of a unilateral transverse histologic slide at the height of the thalamic and geniculate nuclei from the formalin-fixed, paraffin-embedded brains of 4 female LR dogs (Supplemental Table S1; cases 6, 12, 14, and 16). All dogs had numerous intracytoplasmic neuronal inclusions of comparable histologic morphology in the section based on hematoxylin and eosin staining. One dog had neurologic signs, and 3 were control dogs euthanized for other reasons (Supplemental Table S1). The periodic acid-Schiff (PAS) reaction along with PAS-diastase treatment was used to detect glycogen and glycoproteins. Luxol fast blue and oil red O were used to identify lipids and lipoproteins. Phosphotungstic-acid-hematoxylin (PTAH) staining was used to reveal arginine-, lysine-, and histidine-rich substances. Bielschowsky’s silver staining was used to detect argyrophilic aggregates. Masson’s trichrome stain was used to detect fibrinous inclusions. Antigens were retrieved at pH 6 with 0.01 M citrate buffer, and the sections were heated at 99°C for 20 minutes for immunohistochemistry with antibodies against ubiquitin (ab 7780, Abcam rabbit polyclonal, Cambridge, UK), LC3B (ab48394, Abcam rabbit polyclonal, Cambridge, UK), p62/SQSTM1 (P0067, Sigma-Aldrich, rabbit polyclonal, Darmstadt, D), and α-synuclein (5G4, Roboscreen, mouse monoclonal, Leipzig, D). For Aβ IHC (4G8, Biolegend, mouse monoclonal, San Diego, USA), sections were pretreated with formic acid for 5 minutes. An UltraVision Detection System HRP/DAB kit (Thermo Fisher Scientific Inc., Waltham, USA) was used to reveal the signal. Positive control material included brain slides from an LR dog with genetically confirmed vacuolar storage disease (p62, ubiquitin, and LC3), a Tibetan spaniel with clinically confirmed canine cognitive decline (amyloid precursor protein for β-amyloid), and a Chihuahua with neuroaxonal dystrophy (α-synuclein). A negative control slide without the primary antibody was included for each antibody. Immunolabeling in inclusion bodies was considered positive, whereas the absence of imimunolabeling in inclusions was considered a negative result.

### Electron Microscopy

Electron microscopy samples from the lateral geniculate nucleus of 2 LRs (cases 6 and 12) were fixed in 2.5% glutaraldehyde, postfixed with 1% osmium tetroxide, stained with 8% uranyl acetate in 0.69% maleic acid, and embedded in epoxy resin. Ultrathin sections were mounted onto copper grids, stained with Reynolds lead citrate, and viewed with a Jeol JEM-1400 (Jeol Ltd., Tokyo, Japan) electron microscope equipped with a Gatan Orius SC 1000B bottom-mounted CCD-camera (Gatan Inc, Pleasanton, California) at 80 KV.

### Quantification and Topography of Inclusion Bodies

**Figure 1.**
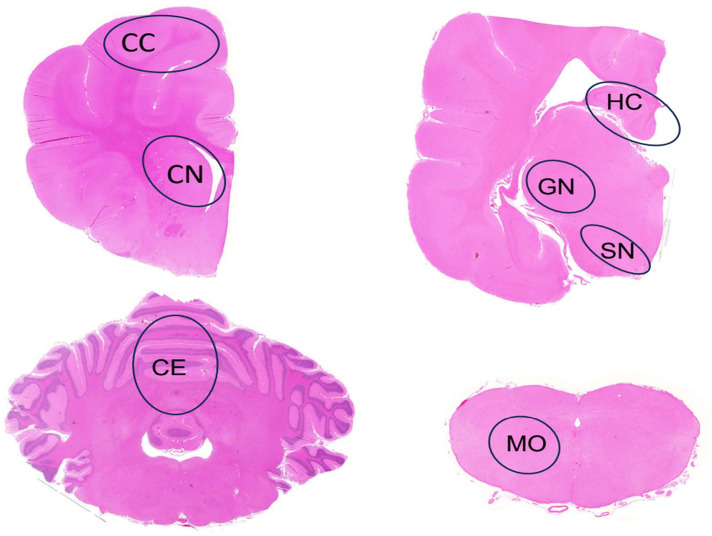
Brain, Lagotto Romagnolo dog. Cross-sections of brain at the different levels evaluated for the presence and number of inclusion bodies. The evaluated areas are outlined in black circles. CC, cerebral cortex; CN, caudate nucleus; GN, geniculate nucleus; HC, hippocampus; SN, substantia nigra; CE, cerebellum; MO, medulla oblongata. Hematoxylin and eosin.

Transversal unilateral sections of 7 brain areas presented in Fig. 1 were examined in all LRs for the presence (1) or absence (0) of intraneuronal inclusions using hematoxylin and eosin-stained sections.

For quantification of inclusions in specific brain regions, 6 age- and gender-matched LRs with and 6 without neurologic signs were further evaluated at the central nervous system levels outlined in Fig. 1. A total of 100 neurons were marked. The number of neurons containing inclusions among the marked cells was counted using Fiji Image J Software 2.0.0 on histologic images acquired with a Zeiss Axio Imager 2 microscope through a 40× objective, using a Zeiss AxioCam MRc5 camera and Zeiss Zen Software (Carl Zeiss Microscopy GmbH, Jena, Germany).

The data analyzed in this study are available upon request from the corresponding author.

### Statistical Tests

The material and results of the quantifications were processed and analyzed using Microsoft Office Excel 2018 (version 16.16.18) and SPSS analysis tool (IBM SPSS Statistics, version 25, Chicago, USA). Cross-tabulation was used to assess the association between neurologic signs and the presence of inclusions in general and in specific brain areas of the dogs. Fisher’s exact test was used to test the statistical significance of the cross-tabulation results. Confidence intervals of 95% were defined using the Wilson method^7^ in Epitools (https://epitools.ausvet.com.au/ciproportion).^22^ The Mann-Whitney *U*-test was used to test the differences in site-specific inclusion body amount between the evaluated brain regions, as the distribution of site-specific inclusion body numbers was skewed. The alpha level was set at 0.05 and was corrected for type 1 error false discovery using the Benjamini-Hochberg method,^4^ as comparison was made between several variables.

## Results

Of 35 LRs autopsied during 2012 to 2018, 23 cases fulfilled the inclusion criteria for this study regarding available anamnestic and clinical data and postmortem material. Among these 23 LRs, 8 LRs presented with neurological signs and 15 did not. The median age of these 23 LRs was 7.5 years (range = 1-16 years), with 14 female and 9 male dogs. Demographic data of the dogs included in this study are presented in Supplemental Table S1. The cohorts for quantification of inclusion bodies consisted of 6 age- and gender-matched LRs with (cases 7, 10, 11, 15, 16, and 22; median age 9.2 years, range = 7-14 years) and without (cases 6, 12, 14, 18, 19, and 23; median age 9.5 years, range = 6-11 years) neurological signs.

### Inclusion Bodies Consist of Proteinaceous Material Rich in Basic Amino Acids

**Figure 2.**
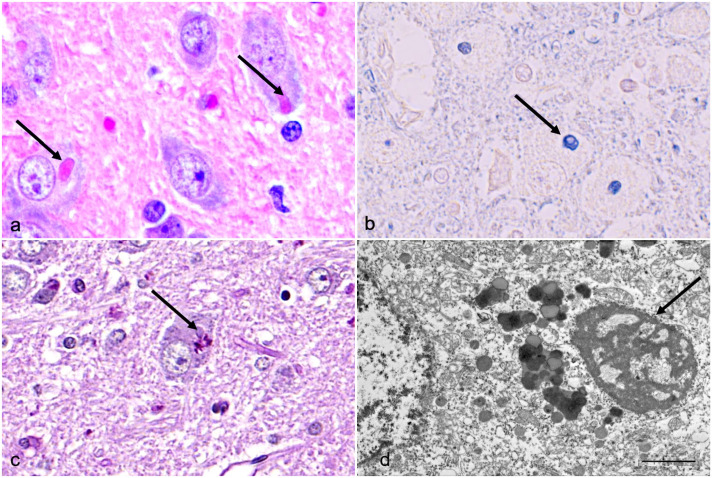
Brain, geniculate nucleus, Lagotto Romagnolo dog. Histochemical and ultrastructural properties of the neuronal inclusions. (a) Cytoplasmic inclusions (arrows) are brightly eosinophilic, well defined, and smooth. Hematoxylin and eosin. (b) Inclusions (arrow) stain deeply basophilic with phosphotungstic-acid-hematoxylin. (c) No periodic acid-Schiff (PAS)-positive reaction is seen in the inclusions (arrow), whereas cytoplasmic lipofuscin serves as a PAS-positive internal control in the neuron. PAS. (d) The inclusions (arrow) are not membrane-bound and consist of electron-dense and finely granular material including small vesicular profiles. Transmission electron microscopy. Bar 2 µm.

The cytoplasmic inclusions evaluated in this study were round to oval, approximately 2 to 8 µm in diameter, with a smooth but undulating surface, and stained brightly eosinophilic with hematoxylin and eosin (Fig. 2a). The inclusions stained positively with PTAH in all tested samples (Fig. 2b). The PTAH is a histochemical stain with a high affinity to the basic amino acids lysine, arginine, and histidine. The PAS reaction in the inclusions (Fig. 2c) and histochemical staining for lipids and fibrin were negative. The inclusions were not argentophilic in the Bielschowsky reaction. In electron microscopy, the inclusion material was electron dense and finely granular with some small vesicular profiles without a limiting membrane (Fig. 2d). No p62, LC3, or ubiquitin immunolabeling was detected within the inclusions, indicating that they are not part of autophagic or proteasomal degradation. Aggregation of α-synuclein or β-amyloid was also not detected within the inclusions. Immunohistochemical results are presented in Supplemental Figure S1.

### Cerebral Cortical Inclusions are Significantly Associated With the Absence of Neurological Signs, Whereas the Overall Presence and the Total Burden of Inclusion Bodies Is Not

**Figure 3.**
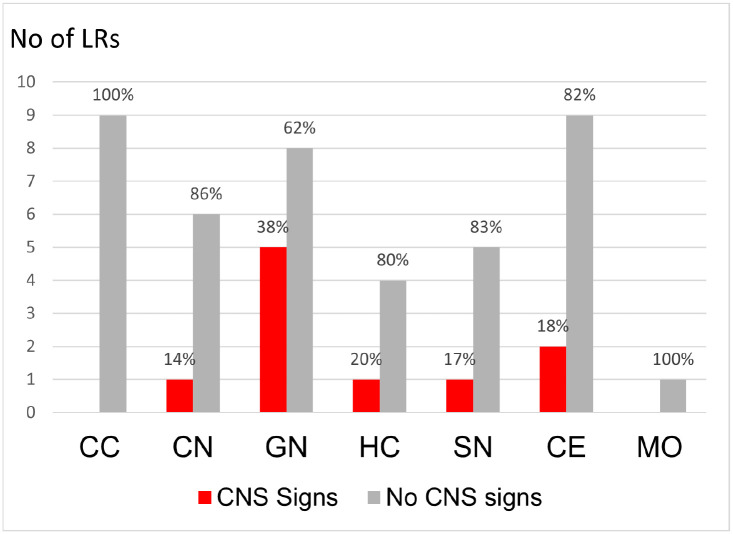
Bar charts showing the distribution of inclusion bodies throughout the neuraxis in symptomatic (CNS signs, red) and asymptomatic (no CNS signs, light gray) Lagotto Romagnolo dogs (LR). The Y-axis shows the total number of LRs with inclusion bodies at the site specified on the X-axis. The percentages of symptomatic and asymptomatic dogs with inclusions at each specific site are marked at the bar. CC, cerebral cortex; CN, caudate nucleus; GN, geniculate nucleus; HC, hippocampus, SN, substantia nigra; CE, cerebellum; MO, medulla oblongata.

Neuronal cytoplasmic inclusion bodies were present in most autopsied LRs (19/23). The overall presence or absence of inclusion bodies was not significantly associated with the dogs’ neurologic status in this study (*P* = .589). Inclusion body prevalence was 87% in LRs without neurologic signs (13/15) and 75% in LRs with neurologic signs (6/8). Inclusions were present in 50% of the dogs aged ≥8 years and in 44% of those aged <8 years (4/9). There was no significant association between senescence and inclusion body presence at a 95% degree of confidence (*P* = .07). Inclusion bodies were most often found in neurons of the lateral geniculate nuclei (13/23), the thalamic nuclei (13/23), and the cerebellar Purkinje cells (11/23). When comparing the distribution of inclusion bodies along the neuraxis with the absence of signs in the dog, only asymptomatic LRs presented with inclusion bodies in neurons of the cortex of the cerebrum (9/23). Fig. 3 presents the number of dogs with neuronal inclusions at specific sites along the neuraxis and the dogs’ neurologic status. The presence of inclusions in the cerebral cortex was significantly associated (*P* = .007) with the absence of signs and remained so after correcting for multiple comparisons (*P* = .056). Significant differences in site-specific number of inclusion bodies were not detected when comparing the quantitative results from 6 LRs with neurologic signs to 6 asymptomatic LRs (*P* = .310).

## Discussion

This study revealed that PTAH-positive cytoplasmic neuronal inclusions are a common histologic finding in the brain of LRs, especially in the thalamus, geniculate nuclei, and cerebellar cortical Purkinje cells, regardless of neurologic status. Neurologically asymptomatic LRs tended to have more inclusion bodies and a more widespread distribution of inclusion bodies along the neuraxis than LRs with neurological signs. Interestingly, the presence of inclusions specifically in the cerebral cortex was significantly associated with the absence of neurological signs in LRs in this study.

Non-infectious, incidental neuronal inclusion bodies have previously been reported in dogs, with some differences in comparison with the findings in LRs. Inclusions were located mainly in the lobus piriformis, substantia nigra, and pontine nuclei in previous studies, whereas the thalamus and the cerebellar cortex were most consistently affected in LRs.^25,28^ Ultrastructural features and composition of the previously described incidental neuronal inclusions in dogs were also different from those of LRs. Suzuki et al reported PAS-positive material containing microtubules located within the endoplasmic cisternae. In contrast, the inclusions of LRs were PAS-negative and free in the cytoplasm.^25^ The neuronal inclusions in previous reports were also larger than those of the LRs, with size extending to 40 μm in diameter.^25^ Based on ultrastructural findings and spatial distribution, inclusions in canine thalamic neurons reported by Nietfeld et al were morphologically more comparable to those in LRs. However, the histochemical properties were not further investigated.^20^ Neuronal inclusion bodies described in conjunction with inflammatory changes in the brain affected neurons in sites more comparable to those of the LRs, including Purkinje cells.^8,13^ An infectious viral etiology was excluded in those cases and was also originally considered and excluded in LRs.^15^ The electron microscopy findings of this study further support the conclusion that these inclusion bodies are noninfectious. Recently, noninfectious inclusion bodies morphologically comparable to Negri bodies and of a similar composition as the inclusions in neurons LRs were reported in thalamic neurons of a red kangaroo and in C57BL/6J mice.^18,27^

The etiology of the neuronal inclusions in LRs is unknown. Similar murine and canine neuronal inclusions have been interpreted as age-related and possibly accelerated by neurologic stress and degeneration.^11,25,27,30^ In a study by Suzuki et al, 80% of the dogs with inclusion bodies were >8 years old. As neurodegeneration starts as early as the sixth year of life in the dog, >8 years of age is often used to define aged canines.^23^ The prevalence of inclusion bodies in the LRs studied here was not significantly associated with senescence. The incidental neuronal inclusion bodies coinciding with inflammatory changes in the canine brains of the previous studies also affected younger dogs at 4 and 6 years of age.^8,13^ Based on the PAS and immunohistochemistry results in this study, the neuronal inclusions of LR did not contain disease-specific proteins observed in known proteinopathies of dogs and humans, such as polyglucosan bodies in Lafora disease, amyloid protein in human AD, and α-synuclein in PD.^9^

We assessed the association between the presence of inclusions, distribution of inclusion bodies along the neuraxis, and site-specific amount of inclusion bodies with the neurologic status of the LR, as inclusion bodies (such as lipofuscin) can be incidental, age-related, or disease-associated depending on the amount and extent of storage.^28,29^ The overall presence of inclusion bodies was not associated with the dog’s neurologic status in LRs, indicating that inclusion bodies of this type may be an incidental finding, consistent with previous reports.^8,11,13,18^ Interestingly, there was a tendency for asymptomatic LRs to have more inclusions. Significantly, only asymptomatic LRs also had inclusion bodies in the cerebral cortex. This may indicate that extensive inclusion body formation along the neuraxis reaching the cerebral cortex protects LRs from neurologic signs. Although inclusion bodies often contain disease-specific protein and are considered part of the pathogenesis in several neurodegenerative diseases, a possible protective role of inclusion body formation has also been recognized.^3,10,32^ Aggregated protein that stresses the neuron and cannot efficiently be degraded by the proteasome can be sequestered in inclusion bodies, thus protecting the cell. Inclusions may then be delivered to the paranuclear aggresome and await degradation as inclusions by mechanisms more efficient than proteasomal degradation, namely autophagy and lysosomal degradation.^10^ When selecting the sites to evaluate for the presence of inclusions in LRs, we chose approximate locations along the neuraxis according to disease staging of AD and PD in humans.^5,6^ In AD and PD, histopathologic findings of neurodegeneration proceed from deeper brain areas, including the dorsal motor nucleus of vagus, via substantia nigra and striatum in PD, and the entorhinal cortex and the limbic system in AD, to ultimately reach the cerebral cortex. Braak suggested that proteinaceous material can be transported transsynaptically and propagate neurodegeneration within the neuraxis and proposed disease staging according to this pattern for AD and PD.^5,6^ In comparison, inclusion body formation could be viewed as a protective process or response in neurons of LRs, as the presence of inclusions in the cerebral cortex was noted more often in LR without signs than in LRs with neurologic signs. To test this hypothesis, investigations comparing neuronal stressors in asymptomatic LRs with and without inclusions would be needed to identify triggers of inclusion formation.

Several factors not specifically assessed in this study, such as gender, inbreeding, the dog’s environment, and lifestyle may affect the formation and distribution of inclusion bodies. Although quantification of inclusions was performed on age- and gender-matched female cohorts to minimize the effects these factors, this also prevents generalization of results to males. The heterozygosity of the Finnish LR breed (29.4%, range 17.3%-33.7%) was not significantly lower than that of LRs in general (29.6%, range = 17.9%-34.1%) or than the general genetic diversity of dog breeds (28.8% range = 15.3%-40.1%), indicating that inclusion formation may not be linked to inbreeding.^24^ All LRs included in this study were private pet dogs. However, more detailed information on activity level, lifestyle, and living environment of the dogs was not available for this study.

In conclusion, PTAH-positive proteinaceous cytoplasmic neuronal inclusion bodies are a common postmortem finding in LRs that is unrelated to amyloid or synuclein aggregation or disturbed protein degradation. The presence of inclusion bodies in the cerebral cortex was associated with the absence of neurologic signs in LRs. This may suggest that inclusions distributed this far along the neuraxis protect the dogs from signs. However, inclusions were also common in other areas of the central nervous system in LRs with neurologic signs. Therefore, inclusion body formation in general does not seem to be a protective response in this breed, but rather an incidental finding.

## Supplemental Material

sj-pdf-1-vet-10.1177_03009858241300555 – Supplemental material for Neuronal cytoplasmic inclusion bodies in the brain of Lagotto Romagnolo dogs: A qualitative and quantitative histologic evaluation Supplemental material, sj-pdf-1-vet-10.1177_03009858241300555 for Neuronal cytoplasmic inclusion bodies in the brain of Lagotto Romagnolo dogs: A qualitative and quantitative histologic evaluation by Sini Peura, Elina Kiiskinen, Tarja S. Jokinen, Anna-Maija K. Virtala and Pernilla Syrjä in Veterinary Pathology
