# Supplementary material for: Neuronal cytoplasmic inclusion bodies in the brain of Lagotto Romagnolo dogs: A qualitative and quantitative histologic evaluation
Source: Vet Pathol. 2024 Dec 9;62(4):539–45. doi: 10.1177/03009858241300555 (PMC12185902; doi:10.1177/03009858241300555)
Supplement: sj-pdf-1-vet-10.1177_03009858241300555 – Supplemental material for Neuronal cytoplasmic inclusion bodies in the brain of Lagotto Romagnolo dogs: A qualitative and quantitative histologic evaluation [file sj-pdf-1-vet-10.1177_03009858241300555.pdf]

Supplemental Materials

Neuronal cytoplasmic inclusion bodies in the brain of Lagotto Romagnolo dogs: a qualitative and quantitative histologic evaluation

Sini Peura, Elina Kiiskinen, Tarja S. Jokinen, Anna-Maija K. Virtala, Pernilla Syrjä

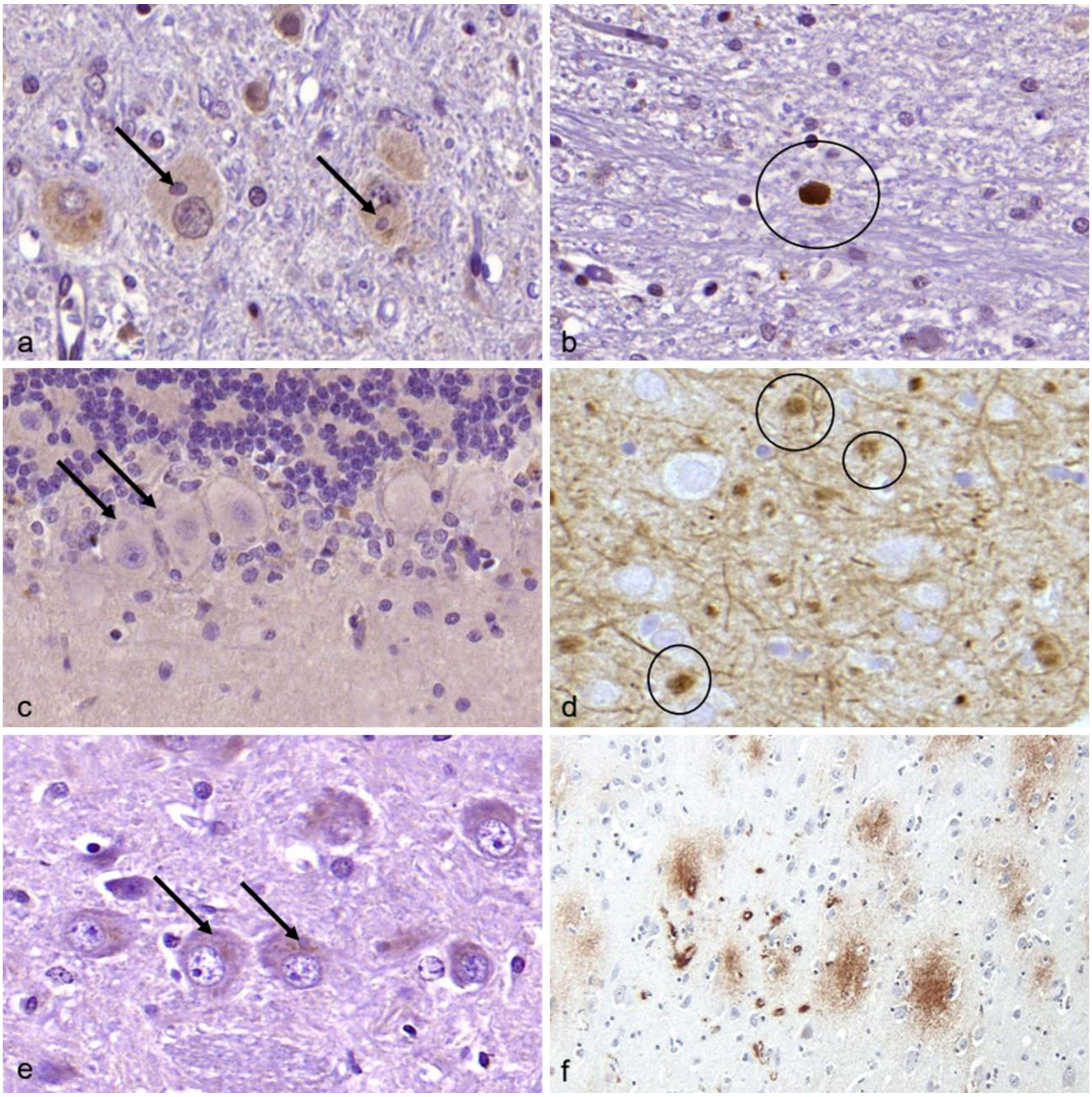

Supplemental Figure S1. Brain, Lagotto Romagnolo (LR) dog. Immunohistochemical findings in the neuronal cytoplasmic inclusion bodies (arrows) of LR dogs. (a) The inclusions do not contain p62, an autophagic cargo marker. p62 immunohistochemistry (IHC). (b) Positive p62 immunolabeling in an axonal spheroid (circled) of a *ATG4D*$^{mut/mut}$ LR with vacuolar storage disease. p62 IHC. (c) Alpha-synuclein is not accumulating in the inclusions in LRs. Alpha-synuclein IHC. (d) Alpha-synuclein immunolabeling in spheroids (circled) of a Chihuahua affected by neuroaxonal dystrophy. Alpha-synuclein IHC. (e) The inclusions of LRs do not contain amyloid. Amyloid precursor protein (APP) IHC. (f) APP immunolabeling in the cerebral cortex and vascular walls of a 16-year-old LR.

Supplemental Table S1. Demographic data regarding the 23 Lagotto Romagnolo dogs included in the study of neuronal inclusion.

Case	Gender	Age years	Neurologic signs	Inclusions	Clinical findings/ cause for euthanasia	Post mortem CNS findings
1	male	1	yes	yes	aggression, decreased menace, seizures	cerebellar cortical degeneration
2	male castrated	2	yes	yes	drooling, regurgitation, aggression, ataxia,	no specific CNS findings
3	male	3	no	no	pulmonary hemangiosarcoma	no specific CNS findings
4	male	5	no	no	hemorrhagic bowel syndrome	no specific CNS findings
5	male castrated	5	yes	no	aggression, weakness	no specific CNS findings
6	female spayed	6	no	yes	osteoarthrosis	no specific CNS findings
7	female	7	yes	no	ataxia	cerebellar cortical degeneration
8	male castrated	7	no	no	acute hemoabdomen	no specific CNS findings
9	male castrated	7	no	yes	severe audiophobia	no specific CNS findings
10	female	8	yes	yes	intetion tremor, ataxia	cerebellar cortical degeneration
11	female	8	yes	yes	epilepsy	rare axonal spheroids
12	female spayed	9	no	yes	osteoarthrosis	no specific CNS findings
13	male	10	no	yes	osteoarthrosis	no specific CNS findings
14	female spayed	10	no	yes	osteoarthrosis	no specific CNS findings
15	female	10	yes	no	canine cognitive dysfunction	rare axonal spheroids
16	female spayed	10	yes	yes	ataxia and one epileptic seizure	no specific CNS findings
17	male	10	no	no	spontaneous death	no specific CNS findings
18	female	11	no	no	widespread tumor	no specific CNS findings
19	female	11	no	no	heart failure, pneumonia	no specific CNS findings
20	female	12	no	no	tumor in the throat	no specific CNS findings
21	female spayed	13	no	yes	fever and diarrhea	no specific CNS findings
22	female spayed	14	yes	no	epilepsy	no specific CNS findings
23	female	16	no	no	mammary tumors and mastitis	no specific CNS findings

Abbreviations: CNS, central nervous system.
